# Temozolomide hexadecyl ester targeted plga nanoparticles for drug-resistant glioblastoma therapy via intranasal administration

**DOI:** 10.3389/fphar.2022.965789

**Published:** 2022-08-17

**Authors:** Siqi Wang, Yawen Yu, Aiping Wang, Xinliu Duan, Yuchen Sun, Liangxiao Wang, Liuxiang Chu, Yanan Lv, Nan Cui, Xuesong Fan, Chunjie Sha, Lixiao Xu, Kaoxiang Sun

**Affiliations:** ^1^ School of Pharmacy, Key Laboratory of Molecular Pharmacology and Drug Evaluation (Yantai University), Ministry of Education, Collaborative Innovation Center of Advanced Drug Delivery System and Biotech Drugs in Universities of Shandong, Yantai University, Yantai, China; ^2^ State Key Laboratory of Long-Acting and Targeting Drug Delivery System, Luye Pharmaceutical Co, Ltd, Yantai, China

**Keywords:** glioblastoma, drug resistance, temozolomide hexadecyl ester, anti-EphA3, nanoparticles, intranasal administration

## Abstract

**Introduction:** Temozolomide (TMZ) is the first-line drug for glioblastoma (GBM), but it is limited in clinical use due to the drug resistance, poor brain targeting, and side effects. Temozolomide hexadecyl ester (TMZ16e), a TMZ derivative with high lipophilicity, membrane permeability, and high anti-glioma properties, has the potential to reverse drug resistance. In this study, anti-ephrin type-A receptor 3 (EphA3) modified TMZ16e loaded nanoparticles (NPs) were prepared for targeted GBM therapy via intranasal administration to deliver TMZ16e to the brain, treat drug-resistant glioma effectively, and reduce peripheral toxicity.

**Methods:** TMZ16e loaded NPs were prepared by emulsion solvent evaporation method followed by modified with anti-EphA3 (anti-EphA3-TMZ16e-NPs). *In vitro* evaluations were performed by an MTT assay and flow cytometry analysis. The orthotopic nude mice models were used to evaluate the anti-glioma effect *in vivo*. Additionally, we investigated the anti-drug resistant mechanism by western blot analysis.

**Results:** The particle size of the prepared NPs was less than 200 nm, and the zeta potential of TMZ16e-NPs and anti-EphA3-TMZ16e-NPs were -23.05 ± 1.48 mV and -28.65 ± 1.20mV, respectively, which is suitable for nasal delivery. *In vitro* studies have shown that anti-EphA3 modification increased the cellular uptake of nanoparticles in T98G cells. The cytotoxicity in the anti-EphA3-TMZ16e-NPs treated group was significantly higher than that of the TMZ16e-NPs, TMZ16e, and TMZ groups (*p* < 0.01), and the cell cycle was blocked. Western blotting analysis showed that the TMZ16e-loaded NPs were able to effectively downregulate the expression level of O6-methylguanine-deoxyribonucleic acid-methyltransferase (MGMT) protein in T98G cells and reverse drug resistance. *In vivo* studies showed that the median survival time of tumor-bearing nude mice in the anti-EphA3-TMZ16e-NPs group was extended to 41 days, which was 1.71-fold higher than that of the saline group and the TUNEL staining results of the brain tissue section indicated that the TMZ16e-loaded NPs could elevate apoptosis in T98G cells.

**Conclusion:** In conclusion, the TMZ16e-loaded NPs can be effectively delivered to the brain and targeted to gliomas, exhibiting better anti-glioma activity, indicating they possess great potential in the treatment of drug-resistant glioma.

## 1 Introduction

Glioblastoma multiforme (GBM) is the most common primary brain tumor with an extremely poor prognosis (Louis et al., 2016). Patients with advanced glioma have a median survival time of only 8–10 months (Stupp et al., 2005), and the 5-years mortality rate exceeds 95% (Stupp et al., 2009). Surgical resection and adjuvant or neoadjuvant chemotherapy are currently the standard treatments for GBM (Kotliarova and Fine, 2012; Gao et al., 2014; Svec et al., 2018). Temozolomide (TMZ) is an alkylating chemotherapeutic agent that is used to treat GBM as the first-line therapy (Hao et al., 2017; Li et al., 2020). However, O^6^-methylguanine-DNA methyltransferase (MGMT) inhibits the anti-glioma activity of TMZ (Ochs and Kaina, 2000; Ding et al., 2020). MGMT is a DNA repair protein that is overexpressed in glioma cells and can repair the TMZ-induced DNA damage, resulting in TMZ resistance in glioma cells (Liu et al., 1996; Gerson, 2004). Studies have shown that at least 50% of patients treated with TMZ developed chemotherapy resistance (Jaeckle et al., 1998; Zhang et al., 2018).

Our previous studies have synthesized temozolomide hexadecyl ester (TMZ16e), a TMZ derivative with high anti-glioma activity, which increased the lipophilicity and biological half-life of the drug by improving the physicochemical and biological characteristics of TMZ. TMZ16e was concluded to effectively increase the consumption of MGMT, thus reversing TMZ resistance and improving glioma treatment efficacy. Additionally, the broad development prospects and potential for the clinical application of TMZ16e have been verified (Yawen et al., 2021). Therefore, in order to accomplish the delivery of TMZ16e to the brain, TMZ16e was used as a model drug to construct a targeted drug delivery system, aim to overcome TMZ resistance, exert a better anti-glioma activity, and provide a basis for the clinical application of TMZ16e. However, the ineffective transportation of chemotherapeutic agents across the blood-brain barrier (BBB) is the biggest limitation of the treatment of central nervous system (CNS) diseases (Friedman et al., 1998). According to studies, the BBB can prevent most small-molecule drugs and almost all macromolecular drugs from entering the CNS (Gooijer et al., 2015), limiting the treatment of brain tumors. Intranasal administration has been regarded as a non-invasive alternative to directly targeting brain tumors (Jigar et al., 2015; Battaglia et al., 2018), as it allows for direct drug delivery into the brain via the olfactory and trigeminal pathways, bypassing the BBB and avoiding first-pass metabolism (Suppasansatorn et al., 2006; Jeyarama et al., 2016). Therefore, the nasal route not only can exhibit high brain targeting efficiency but also reduce the systemic adverse effects of the drugs, thus improving medication adherence in patients (Martins et al., 2019; Agrawal et al., 2020).

Nowadays, poly (lactide-co-glycolide) nanoparticles (PLGA NPs) are widely used in nasal drug delivery systems. Studies have shown that PLGA NPs are preferable to other polymeric NPs for the brain-targeted delivery of drugs through the nasal route (Illum, 2000). Indeed, in the case of nose-to-brain delivery, NPs can increase the transcellular transport of encapsulated drugs through the neuroepithelium, thus increasing drug availability in the brain (Boyd et al., 1992; Pasquale, 2008; Day et al., 2013). Nose-to-brain delivery has been an attractive strategy for the treatment of diseases with CNS due to its high delivery efficiency. Studies have shown that Perillyl alcohol (POH) (Fonseca et al., 2011), glioma-adapted vesicular stomatitis virus strain (VSVrp30) (Fultz et al., 1982) and methotrexate (Shingaki et al., 2010) have been successfully targeted brain tumors by nasal route. Additionally, Danielyan L et al. (Jiang et al., 2011) investigated nasal delivery for the complex surgical procedure of stem cell transplantation in Parkinson’s disease, which confirmed that the nasal delivery was a reliable alternative strategy. Therefore, the nasal administration of TMZ-loaded NPs was hypothesized to be a feasible strategy for enhancing the therapeutic efficacy of TMZ16e and overcoming TMZ resistance. Additionally, studies have shown that the ephrin type-A receptor 3 (EphA3) is a tumor-specific therapeutic target and is widely accepted in GBM treatment (Day et al., 2013). EphA3 is a membrane-associated receptor, which is highly expressed in the tumor-initiating cell population in glioma cells, but lowly in normal cells (Wang et al., 2021). Therefore, the EphA3 antibody (anti-EphA3) as a non-fucosylated IgG1j (human f-allotype) monoclonal antibody with low toxicity can further enhance glioma targeting via its specific interactions with the EphA3 receptor. Our previous studies have designed and developed TMZ short-chain ester derivative TMZ4e (TBE) loaded NPs functionalized with anti-EphA3 for glioblastoma targeting, which showed excellent GBM targeting effect and anti-glioma activity (Chu et al., 2018). In this study, we referred to the advantages of TBE-NPs in brain delivery and constructed TMZ16e-based anti-EphA3-modified NPs, aiming to deliver TMZ16e to GBM efficiently and overcome the challenges of drug-resistant glioma treatment.

In order to deliver TMZ16e to the brain and concentrate it on the glioma site, effectively treat drug-resistant glioma, TMZ16e-loaded NPs were constructed to achieve glioma-targeted drug delivery via the nasal route. Firstly, the physicochemical characteristics of nanoparticles were characterized to evaluate their suitability for nose-to-brain drug delivery. Secondly, the therapeutic efficacy of TMZ16e on drug-resistant glioma cells was assessed *in vitro* and *in vivo* using T98G cells and GBM orthotopic nude mouse models, respectively. Moreover, the protein expression levels of MGMT were used to explore the resistance mechanism by western blot analysis. This study aims to identify potential and feasible treatment strategies for the clinical care of drug-resistant glioma.

## 2 Materials and methods

### 2.1 Materials and animals

TMZ was obtained from Wuhan Fuxin Chemical Co., Ltd. (Wuhan, China). PLGA 5050 2 A (lactide/glycolide ratio: 50/50; molecular weight: 18,000) was provided by Shandong Luye Pharmaceutical Co., Ltd. (Yantai, China). The coumarin-6 was obtained from Shanghai Macklin Biochemical Co., Ltd (Shanghai, China). The EphA3 antibody and the phosphate buffered saline (PBS) were purchased from Sigma-Aldrich (Saint Louis, MO, United States).

The human bronchial epithelial cell lines (16HBE) and human glioma T98G cells lines (T98G) were provided by the American Type Culture Collection (Zhongyuan, Ltd. Beijing, China), which were grown in DMEM supplemented with 10% of fetal bovine serum (FBS) and 1% of penicillin/streptomycin. These cell lines were cultured in a 5% CO2 incubator at 37°C. Male nude mice (5–6 weeks old) were purchased from the Chengdu Dossy Experimental Animals Co., Ltd (Chengdu, China). All animal experiments were approved by the Ethical Committee on Animal Experimentation of Yantai University (Yantai, China).

### 2.2 Preparation and characterization of nanoparticles

The emulsion solvent evaporation technique was used to prepare TMZ16e-NPs. TMZ16e and PLGA (50/50, 2A) were dissolved in the mixed solvent of dichloromethane and acetone (3:2). To obtain the primary emulsion, the mixture was added dropwise to the aqueous polyvinyl alcohol (PVA) solution (1% w/v) in an ice bath with sonication (20% amplitude). Then, the suspension was redispersed into the aqueous PVA solution (0.3% w/v) under stirring overnight to evaporate the organic solvent, and the resultant dispersion preparation was obtained. To prepare anti-EphA3-TMZ16e-NPs, anti-EphA3 was thiolated by the Traut’s reagent for 30 s (the molar ratio of anti-EphA3: Traut’s reagent was 1:20), which was then coupled to concentrated TMZ16e-NPs for 30 s at room temperature under nitrogen (N_2_), followed by stirring for 8 h at room temperature to obtain anti-EphA3-TMZ16e-NPs. The average size and zeta potential of TMZ16e-loaded NPs were determined using a Zetasizer Nano ZS (Malvern Instruments, Malvern, United Kingdom). The morphology of the NPs was obtained using transmission electron microscopy (TEM, Hitachi H-7600, Japan). The antibody conjugation efficiency was evaluated using a bicinchoninic acid protein quantification kit (Yikebaide technology, Beijing, China). The encapsulation efficiency (EE%) and drug loading (DL%) of TMZ16e in the NPs were determined by high performance liquid chromatography (HPLC) (Dikma technologies, Foothill Ranch, CA, United States) using a mobile phase composition of acetonitrile and 0.5% acetic acid-water (95:5) at a flow rate of 1.0 ml/min at 327 nm. The EE% and DL% were calculated as follows.

$$\text{EE\%}=\frac{Total\;mass\;of\;drug\;dose-Mass\;of\;free\;drug}{Total\;mass\;of\;drug}\text{×}100\text{\%}$$ (1)

$$\text{DL\%}=\;\frac{Total\;mass\;of\;drug\;dose-Mass\;of\;free\;drug}{Weight\;of\;NPs}\text{×}100\text{\%}\;$$ (2)

### 2.3 *In vitro* release study

Previous studies have indicated that TMZ16e is more stable at an acidic pH than at alkaline and neutral pHs (Yawen et al., 2021). The *in vitro* release studies of TMZ16e from NPs were performed at pH 5.5 at 37°C, which simulated the tumor micro-environment (Ak et al., 2021). NPs were dialyzed in the dialysis bag (MWCO 14,000 Da) that immersed in phosphate buffered saline (PBS) containing 4% SDS, for 30 days at 37°C on a rocker (100 rpm). A volume of 2 ml of release medium was removed and an equal volume of release medium was added at specific time points. Finally, the samples were collected and analyzed using HPLC to evaluate the cumulative release percentage of TMZ16e.

### 2.4 *In vitro* cytotoxicity studies

The 3-(4,5-dimethylthiazol-2-yl)-2,5-diphenyl tetrazolium bromide (MTT) assay was used to assess the safety of the nasal mucosa and the cytotoxicity of drug-resistant glioma cells. The 16HBE cells (8×10^3^ cells/well) and T98G cells (2×10^3^ cells/well) were incubated for 12h in 96-well plates. Then, 16HBE cells and T98G cells were treated with NPs (0.5–15 μM) for respectively 6 and 72 h. After that, the MTT solution (5 mg/ml) was added in the plates (20 μL per well) and incubated for 4 h at 37°C, then removed the media and replaced with 200 μl dimethyl sulfoxide (DMSO), gently shaken for 15 min at room temperature to ensure the dissolution of MTT-formazan crystals. The spectrophotometric absorbance of the sample was determined at 327 nm using a microplate reader (SpectraMax M2, Molecular Devices, San Jose, CA, United States).

### 2.5 Cellular uptake and intracellular positioning

A TCS-NT confocal microscope (Wetzler, Heidelberg, Germany) was used to examine the cellular uptake of NPs in T98G cells. T98G cells (5×10^4^ cells/well) were seeded in 24-well plates and incubated overnight at 37°C. The cells in pre-incubation groups were then incubated with the medium containing anti-EphA3 (20 μg/ml) for 4 h. After that, the cells were incubated with coumarin-6-loaded nanoparticles and anti-EphA3 modified coumarin-6-loaded nanoparticles (anti-EphA3-coumarin-6-loaded NPs) for 0.5, 1, and 2 h, respectively. The cells were then fixed with 4% paraformaldehyde for 15 min after washing with PBS. To further label the intracellular localization of NPs, T98G cells were incubated with Hoechst 33,342 for 15 min. Lastly, the cells were washed thrice, and analysis was performed using fluorescence microscopy.

### 2.6 Western blot analysis

To assess the expression level of MGMT in T98G cells, western blot analysis was employed. T98G cells (3×10^5^ cells/well in 6-well plates) were incubated for 72 h with NPs (equivalent to 5 μM of TMZ), and the cells were collected. After that, the protein concentration was determined by bicinchoninic acid (BCA) kit. The proteins were loaded in sodium dodecyl sulfate-polyacrylamide gel electrophoresis (SDS-PAGE), transferred onto a polyvinylidene difluoride (PVDF) membrane, blocked in 5% milk for 1 h at room temperature, and then incubated with primary antibodies, anti-human MGMT monoclonal antibody (1:1000) and anti-GAPDH (1:1000). The antibodies were subsequently replaced with horseradish peroxidase-labeled goat anti-rabbit lgG (H + L) (1:1000) antibody, which acted as the secondary antibody. The protein bands were detected using enhanced chemiluminescence (ECL).

### 2.7 Cell cycle

To determine the cell cycle, T98G cells (4×10^5^ cells/well) were incubated in 6-well plates overnight and then treated with saline, TMZ16e-NPs, and anti-EphA3-TMZ16e-NPs (equivalent to 5 μM of TMZ) for 72 h, respectively. Afterward, the culture medium was removed, T98G cells were trypsinized, washed with PBS, fixed in 75% ethyl alcohol overnight at 4°C, and then stained with a mixture of ribonuclease A (RNase A) in PBS (20 μg/ml) and propidium iodide (PI) solution (50 μg/ml) for 30 min to detect the cell cycle. The samples were analyzed using a flow cytometer (BD Biosciences, Franklin Lakes, NJ, United States).

### 2.8 Nude mice orthotopic glioblastoma model

A volume of 5 μl of T98G cell suspension was inoculated with a brain stereotaxic instrument, wherein the concentration of T98G cell was 1×10^5^/μl. Injection location: the right cerebral hemisphere of nude mice (pre-halogen 0.5 mm, paracentric 2.5 mm, skull depth 3.5 mm). The growth of tumor and survival time of tumor-bearing nude mice were observed and recorded every day. The tumor growth was monitored using magnetic resonance imaging (Biospec70/20USR, Bruke, Germany).

### 2.9 *In vivo* anti-glioma activity

To assess the anti-glioma efficacy of NPs *in vivo*, the tumor-bearing nude mice were randomly divided into three groups (15 mice per group): saline group, TMZ16e-NPs group, and anti-EphA3-TMZ16e-NPs group (equivalent to 5 mg/kg TMZ). The mice underwent intranasal administration when the tumor diameter reached about 5 mm. On day 15, mice (5 mice per group) were sacrificed, and the brains were harvested to prepare paraffin sections. The thin-tissue sections were deparaffinized in xylene and rehydrated in decreasing concentrations of ethanol, then washed with PBS and fixed in 4% formaldehyde for 15 min. Finally, the tissue sections were washed with PBS again and detected using a terminal deoxynucleotidyl transferase dUTP nick end labeling (TUNEL) apoptosis detection kit according to the manufacturer’s instructions, and apoptotic cells were examined using Image-Pro Plus 5 (Media Cybernetics, Silver spring, United States). Survival time was monitored and analyzed using the Kaplan−Meier method (10 mice per group). Moreover, the body weight of each group of nude mice was recorded every 5 days since the tumor cells were inoculated until the tumor-bearing nude mice died.

Western blot was used to detect MGMT protein expression *in vivo*. The brain tissues of tumor-bearing nude mice were lysed in RIPA lysis buffer. The separated proteins were then loaded onto SDS-PAGE, transferred onto a PVDF membrane, and treated as described above. An ultra-sensitive ECL kit and ImageJ software were used to detect and analyze the protein expression.

## 3 Results and discussion

### 3.1 Characterization of nanoparticles

**FIGURE 1:**
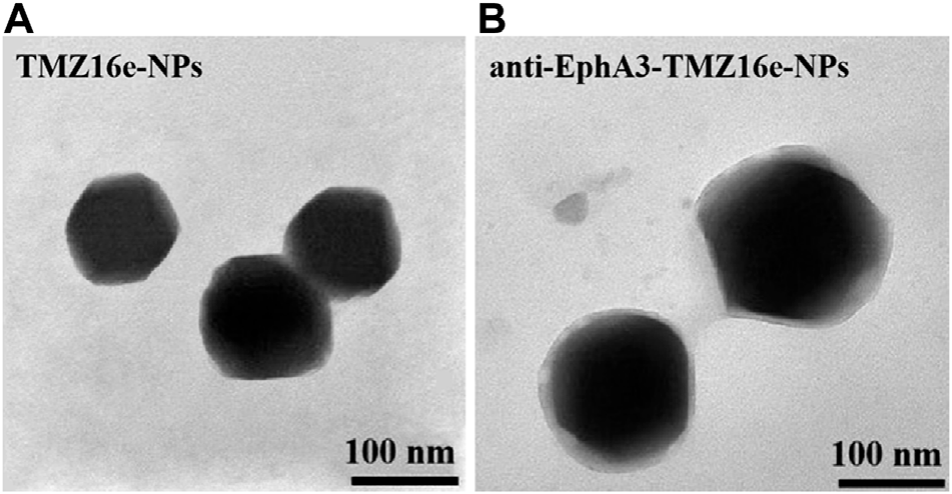
Transmission electron microscope images of **(A)** TMZ16e-NPs and **(B)** anti-EphA3-TMZ16e-NPs.

Figure 1 shows the morphology of TMZ16e-NPs and anti-EphA3-TMZ16e-NPs as observed by TEM. There was no agglomeration of particles and the NPs were spherical with a uniform distribution. The physicochemical properties of TMZ16e-NPs and anti-EphA3-TMZ16e-NPs are summarized in Table 1. The average particle size of the NPs was less than 200 nm, with polydispersity indexes (PDI) values <0.2, indicating that the TMZ16e-NPs were well dispersed and suitable for intranasal administration. The TMZ16e-NPs and anti-EphA3-TMZ16e-NPs had zeta potentials of -23.05 ± 1.48 mV and -28.65 ± 1.20 mV, respectively, indicating NP stability (Mu et al., 2020). The DL% of TMZ16e-NPs and anti-EphA3-TMZ16e-NPs were 17.13% and 16.81%, respectively, and the EE% was more than 90% for both NPs. The conjugation efficiency of anti-EphA3 was 25.12 ± 1.71%.

**TABLE 1:** Characterization of TMZ16e-NPs and anti-EphA3-TMZ16e-NPs (n = 3).

Formulation	Particle size (nm)	PDI	Zeta potential (mV)	Entrapment efficiency (%)	Drug loading (%)
TMZ16e-NPs	121.4 ± 1.8	0.096 ± 0.016	−23.05 ± 1.48	95.72 ± 1.50	17.13 ± 1.01
anti-EphA3-TMZ16e-NPs	135.1 ± 2.4	0.085 ± 0.037	−28.65 ± 1.20	93.58 ± 1.39	16.81 ± 1.17

NPs, nanoparticles; PDI, polydispersity index.

### 3.2 *In vitro* release study

**FIGURE 2:**
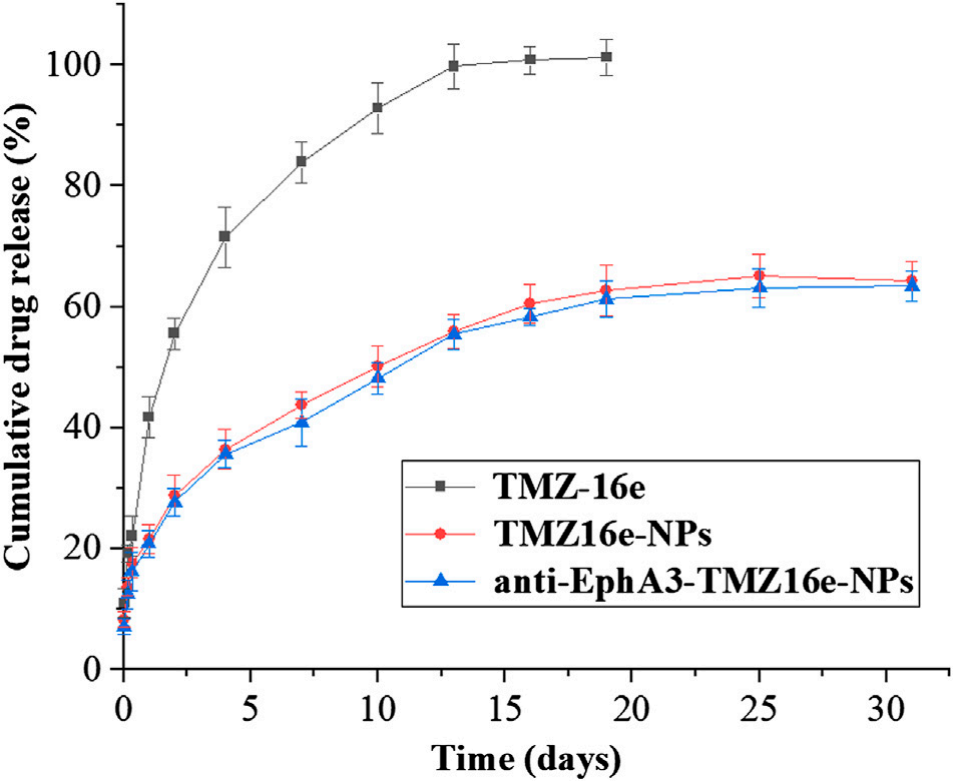
*In vitro* release profiles of TMZ16e-NPs and anti-EphA3-TMZ16e-NPs (*n* = 3).

Figure 2 shows the drug release profiles of TMZ16e-NPs and anti-EphA3-TMZ16e-NPs. The cumulative release of TMZ16e was about 100% after 12 days in pH 5.5 PBS containing 4% SDS, whereas TMZ16e-NPs and anti-EphA3-TMZ16e-NPs released about 63% of TMZ16e in 30 days, indicating that the TMZ16e-loaded NPs had a sustained release profile in the acidic tumor micro-environment.

### 3.3 *In vitro* cytotoxicity studies

**FIGURE 3:**
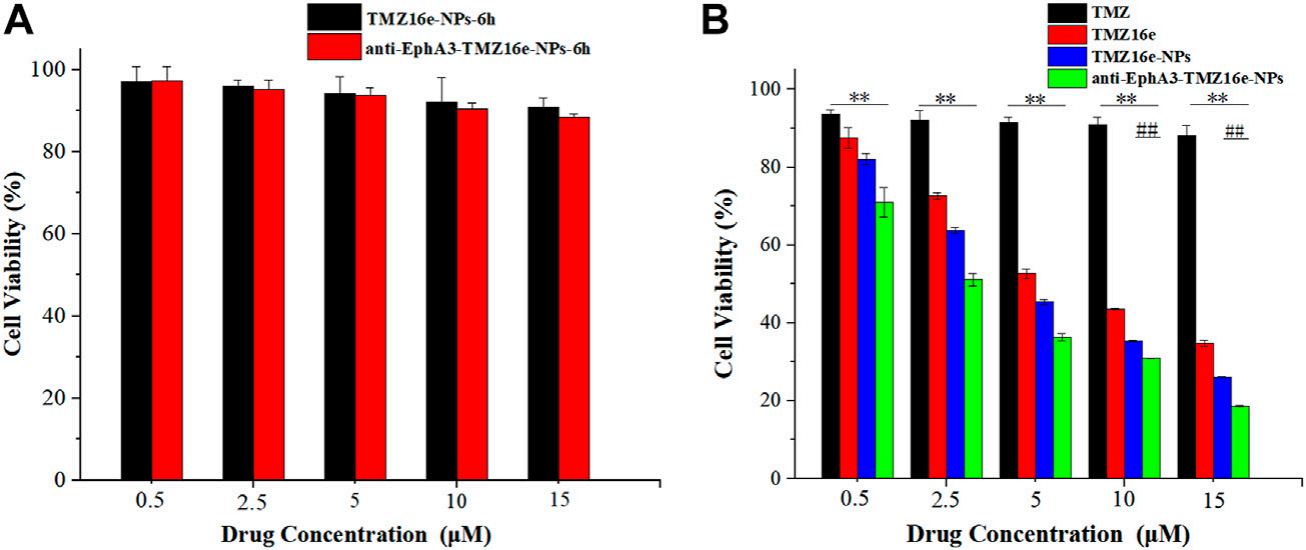
**(A)** Cell viability of 16HBE cells treated with TMZ16e-NPs and anti-EphA3-TMZ16e-NPs for 6 h. **(B)** Cell viability of T98G cells treated with TMZ, TMZ16e, TMZ16e-NPs, and anti-EphA3-TMZ16e-NPs for 72 h (*n* = 3), **p < 0.01 versus TMZ, ^##^ *p* < 0.01 versus TMZ16e-NPs.

Cytotoxicity assay was conducted on the 16HBE cell line to assess the safety of the TMZ16e-loaded NPs on the nasal mucosa. Figure 3A shows that the cell viability of 16HBE cells was greater than 80% even at the highest concentration, indicating that the TMZ16e-loaded NPs are relatively safe. The cytotoxicity of TMZ, TMZ16e, TMZ16e-NPs, and anti-EphA3-TMZ16e-NPs to T98G cells was then studied using the MTT assay. The four formulations inhibited the growth of T98G cells in a concentration-dependent manner, as shown in Figure 3B and Table 2. The calculated IC_50_ value of TMZ16e was 6.08 μM, which was significantly lower than that of TMZ with 703.69 μM (*p* < 0.01), indicating that TMZ16e had a higher anti-glioma activity. According to our previous study, the high anti-glioma activity of TMZ16e may be attributed to its higher lipophilicity (Yawen et al., 2021). The cytotoxicity of TMZ16e on T98G cells was further enhanced after it was encapsulated into NPs, and the half-maximal inhibitory concentration (IC_50_) of TMZ16e-NPs and anti-EphA3-TMZ16e-NPs was 5.36 μM and 2.27 μM, respectively, demonstrating the feasibility of enhancing the cytotoxicity of TMZ16e by encapsulating it into NPs. This could be due to the increased internalization of NPs into cells, resulting in an increase in drug concentration in T98G cells (Bi et al., 2016). In addition, anti-EphA3-TMZ16e-NPs had higher cytotoxicity than TMZ16e-NPs, with a significant difference at concentrations of 10 μM and 15 μM (*p* < 0.01), demonstrating that the anti-EphA3 targeting (Offenhäuser et al., 2018) increased the anti-tumor activity of TMZ16e on drug-resistant glioma cells.

**TABLE 2:** IC^50^ of TMZ, TMZ-16e, TMZ16e-NPs, anti-EphA3-TMZ16e-NPs on T98G cells (n = 6).

Cell line	IC^50^ (μM), 72h			
	TMZ	TMZ-16e	TMZ16e-NPs	anti-EphA3-TMZ16e-NPs
T98G	703.69 ± 0.46	6.08 ± 0.01^a^	5.36 ± 0.23^a^ ^,^ ^b^	2.27 ± 0.37^a^ ^,^ ^b^

a **p < 0.01 versus TMZ.

b ^##^ *p* < 0.01 versus TMZ16e.

### 3.4 Cellular uptake and intracellular positioning

**FIGURE 4:**
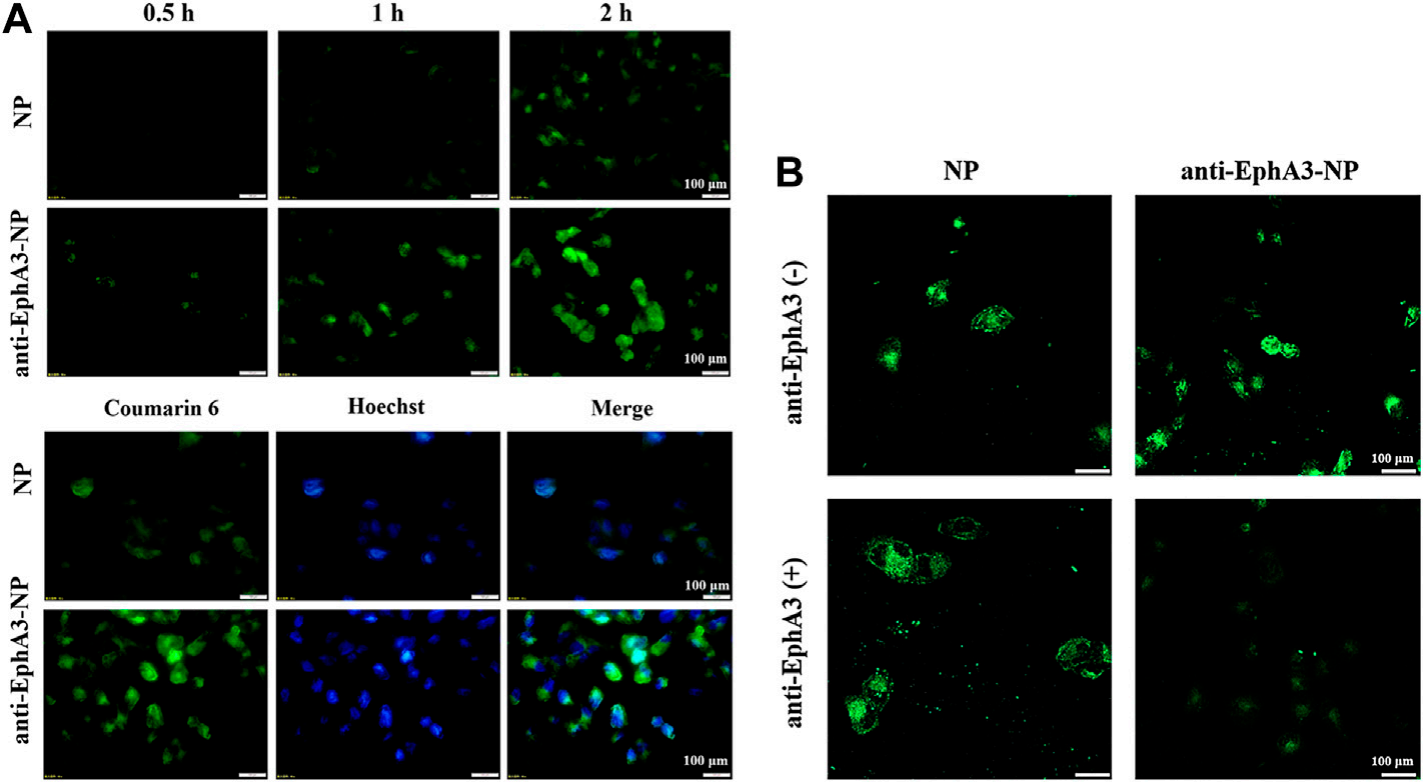
**(A)** Cell uptake and intracellular localization of NPs and anti-EphA3-NPs by T98G cells. **(B)** Cell uptake of NPs and anti-EphA3-NPs with pre-incubated (+) and non-pre-incubated (−) anti-EphA3 by T98G cells.

To assess the high specificity of glioma cells, we next evaluated the cell uptake of coumarin-6-loaded NPs and anti-EphA3-coumarin-6-loaded NPs by confocal microscopy. Figure 4A shows that the cellular uptake of NPs was time-dependent and that the fluorescence intensity in the anti-EphA3-coumarin-6-loaded NPs group was higher than that in the coumarin-6-loaded NPs group, suggesting that the NPs were internalized via endocytosis and that anti-EphA3 targeting contributed to the further cellular internalization of drug-resistant glioma cells. Additionally, as shown in Figure 4B, there was a substantial decrease in the fluorescence signal from anti-EphA3-coumarin-6-loaded NPs groups after preincubation with anti-EphA3, while the coumarin-6-loaded NPs group had no significant change, which implied that anti-EphA3 could competitively restrain the uptake of anti-EphA3-coumarin-6-loaded NPs in T98G cells. All the results indicated that anti-EphA3 modified NPs could increase cellular uptake by binding to the EphA3 receptor specifically, substantiating anti-EphA3 targeting in drug-resistant glioma cells.

**FIGURE 5:**
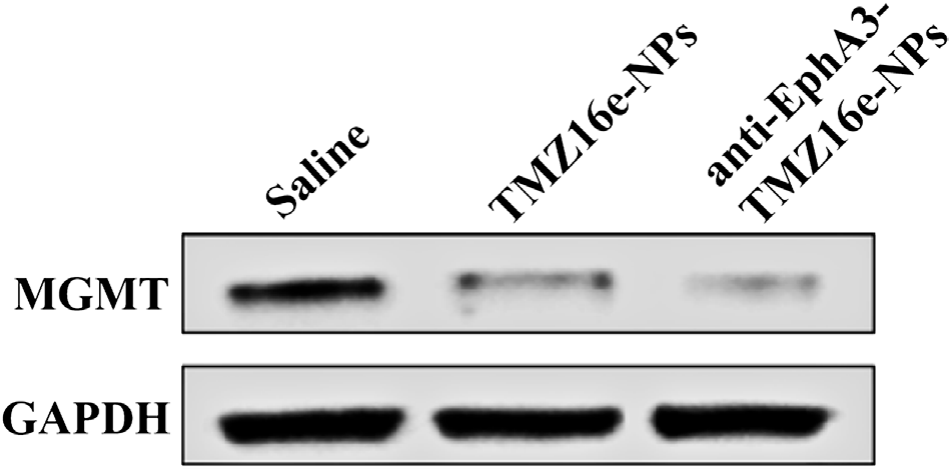
The protein expression level of MGMT and GAPDH in T98G cells treated with TMZ16e-NPs and anti-EphA3-TMZ16e-NPs (*n* = 3).

### 3.5 Western blot analysis

Studies have shown that the overexpression of MGMT may be the key to causing glioma resistance to TMZ (Oldrini et al., 2020). We examined the effect of TMZ16e-loaded NPs on MGMT expression. T98G cells (MGMT+) were incubated with saline, TMZ16e-NPs and anti-EphA3-TMZ16e-NPs for 48 h, and the MGMT levels were detected by western blot. Figure 4 shows that compared with the saline group, the MGMT protein levels were significantly reduced after TMZ16e-NPs and anti-EphA3-TMZ16e-NPs treatment, effectively down-regulating the expression of MGMT. MGMT is a depleting protein (Ochs and Kaina, 2000), so TMZ16e-loaded NPs enhanced chemosensitivity of drug-resistant cells to alkylating agents by depleting more MGMT, thus reversing drug resistance.

### 3.6 Cell cycle

**FIGURE 6:**
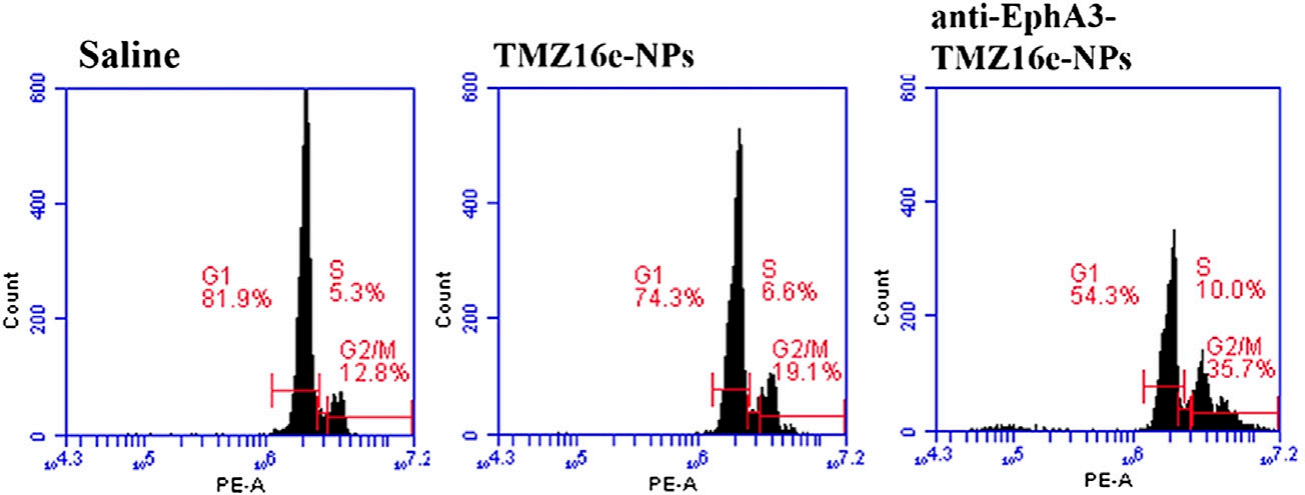
Cell cycle analysis.

Studies have revealed that TMZ led to impaired DNA repair and G2/M arrest through alkylating guanine in DNA at position O^6^, thus exerting the effect of inhibiting glioma cell proliferation (Xie et al., 2018). Flow cytometry was used to assess the cell cycle of the TMZ16e-loaded NPs to acquire a better understanding of the underlying resistance mechanism against T98G cells. The cell cycle in Figure 6 shows that compared to the saline group, T98G cells arrested at the G2/M phase of the cell cycle were increased significantly after treatment with TMZ16e-NPs (19.1% vs. 12.8%), indicating that the TMZ16e had a strong ability to arrest the cell cycle of drug-resistant glioma cells. In addition, compared with TMZ16e-NPs, the percentage of G2/M phase upon anti-EphA3-TMZ16e-NPs treatment increased significantly (*p* < 0.001), which may be due to the modification of the antibody could increase the intracellular concentration of TMZ16e by raising the cellular uptake of NPs in T98G cells, thereby allowing TMZ16e to exert stronger inhibitory effect.

### 3.7 *In vivo* anti-glioma activity

**FIGURE 7:**
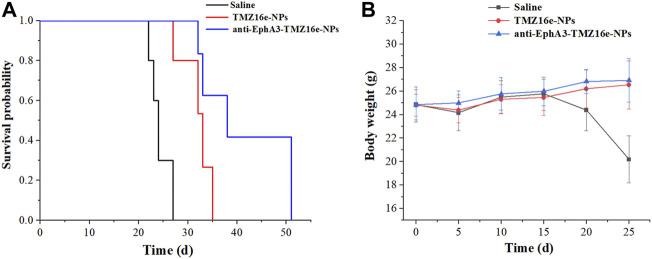
**(A)** Kaplan-Meier survival curve **(B)** Body weight changes of the tumor-bearing nude mice treated with TMZ16e-NPs and anti-EphA3-TMZ16e-NPs (*n* = 10), **p < 0.01 versus saline, ^##^ *p* < 0.01 versus TMZ16e-NPs.

To assess the anti-glioma efficacy of the TMZ16e-loaded NPs *in vivo*, orthotopic glioblastoma tumor-bearing nude mice were used. Figure 7A and Table 3 show that the median survival time of the tumor-bearing nude mice treated with TMZ16e-NPs and anti-EphA3-TMZ16e-NPs was extended to 32 and 41 days, respectively, showing a 1.3 and 1.7-fold increase, respectively, compared to the saline group (*p* < 0.01), showing a significant anti-drug glioma effect. The results demonstrated that TMZ16e-loaded NPs with the proper size and surface chemistry can be used to treat drug-resistant glioma and that anti-EphA3 targeting resulted in stronger inhibition of glioma. Figure 7B shows that in the groups treated with TMZ16e-NPs and anti-EphA3-TMZ16e-NPs, a constant weight gain occurred, also indicating that TMZ16e-loaded NPs could improve anti-glioma activities.

**TABLE 3:** Survival time of tumor bearing nude mice (n = 10).

Groups	Mean (days)	Median (days)	Increase of life span (%)
Saline	24.3	24	−
TMZ16e-NPs	32.1	32**	32.1
anti-EphA3-TMZ16e-NPs	41.4	41**^##^	70.4

**p < 0.01 versus saline.

^##^ *p* < 0.01 versus TMZ16e-NPs.

**FIGURE 8:**
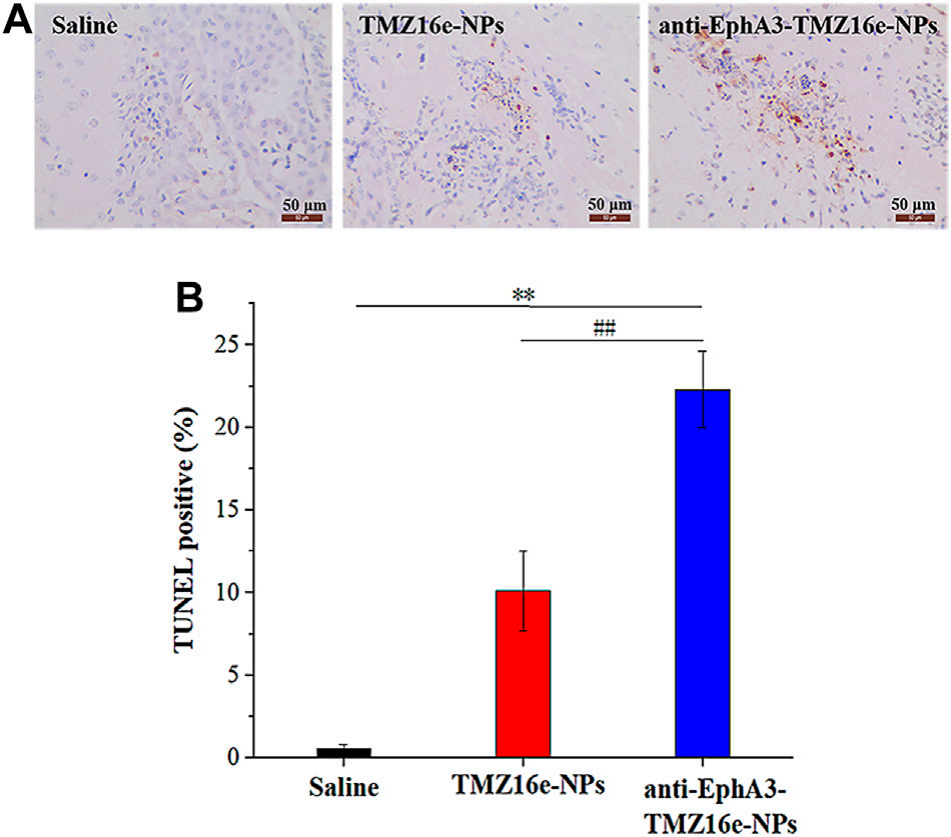
**(A)** TUNEL staining of the brain sections. Brown staining represents apoptotic cells. **(B)** Quantification of TUNEL apoptosis (n = 5), **p < 0.01 versus saline, ^##^ *p* < 0.01 versus TMZ16e-NPs.

The therapeutic effect of the TMZ16e-loaded NPs was further evaluated using histological analysis of brain slices from tumor-bearing nude mice. The TUNEL assay revealed the highest extent of apoptosis in the anti-EphA3-TMZ16e-NPs group (Figure 8A and Figure 8B), and the apoptosis percentages of the TMZ16e-NPs and anti-EphA3-TMZ16e-NPs treatment groups were 10.1% and 22.3%, respectively, which were significantly higher than the saline group (*p* < 0.01). This indicated that the TMZ16e-loaded NPs exerted an anti-glioma activity by inducing cell apoptosis, resulting in a superior therapeutic effect on drug-resistant glioma cells.

**FIGURE 9:**
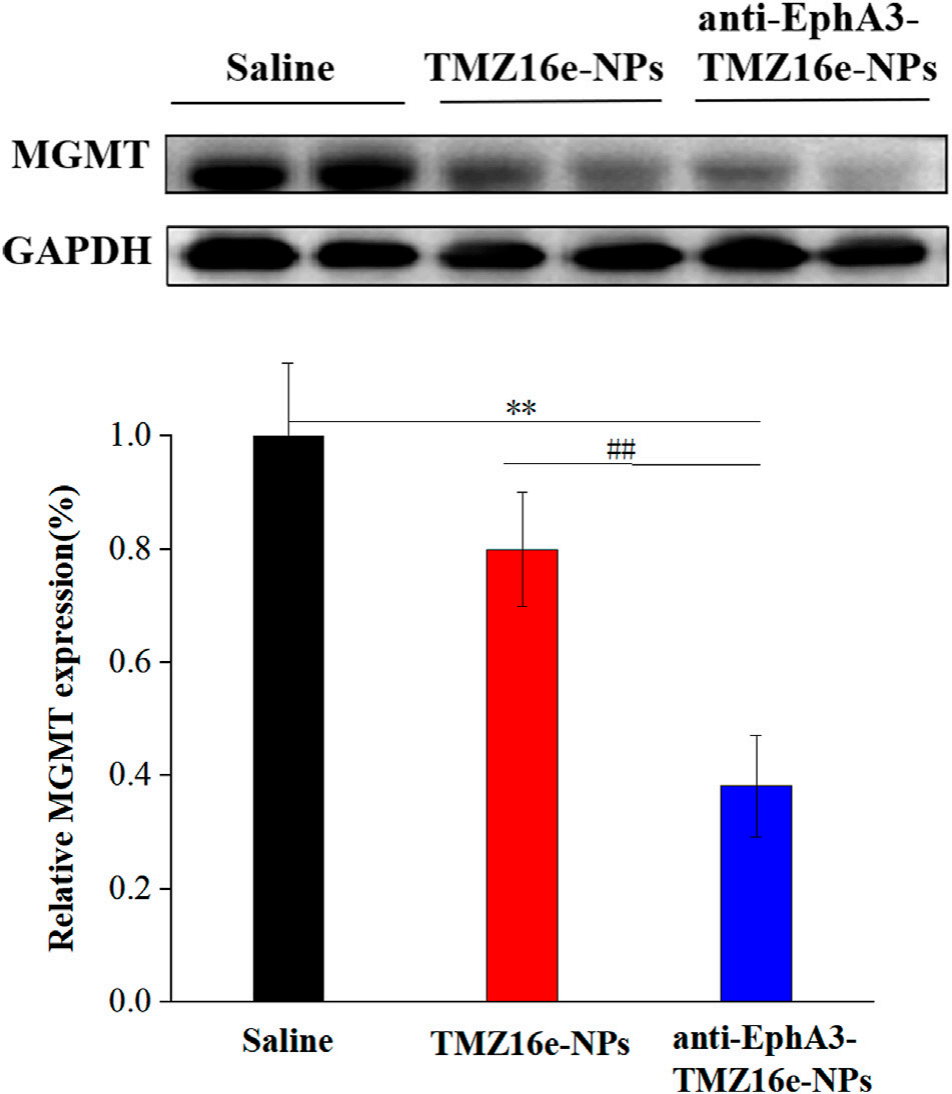
The protein expression level of MGMT and GAPDH in the brain sections of tumor-bearing nude mice treated with TMZ16e-NPs and anti-EphA3-TMZ16e-NPs (*n* = 2), **p < 0.01 versus saline, ^##^ *p* < 0.01 versus TMZ16e-NPs.

Western blot analysis of tumor-bearing nude mice brain sections was used to detect the protein expression level of MGMT. Figure 9 shows that MGMT expression levels were significantly downregulated in the TMZ16e-NPs and anti-EphA3-TMZ16e-NPs groups, whereas they were relatively high in the saline group, demonstrating that TMZ16e-loaded NPs could inhibit the growth of drug-resistant glioma cells by increasing MGMT protein consumption (Wu et al., 2021) and that anti-EphA3 targeting further downmodulated the MGMT protein to reverse TMZ resistance. Thus, the results confirmed the successful delivery of the TMZ16e-loaded NPs in the brain tumor region and its excellent potential in the treatment of drug-resistant glioma.

## 4 Conclusion

TMZ is the mainstay chemotherapeutic drug for glioma. However, its clinical use is limited due to the resistance of glioma cells. Previous studies have confirmed that TMZ16e was superior to TMZ in physicochemical properties, and had the advantages of high anti-glioma activity and reversal of glioma drug resistance. In this study, we further constructed TMZ16e-loaded NPs, and chose intranasal administration to deliver TMZ16e to the brain more effectively and focus on glioma sites, aiming to provide a basis for the clinical application of TMZ16e and a potential strategy for the clinical treatment of drug-resistant gliomas. The physicochemical properties of the TMZ16e-loaded NPs suggested that they could be used in nasal administration. It also increased the cytotoxic potential in T98G cells and inhibited the cell cycle at the G2/M phase. *In vivo* studies in the orthotopic nude mice models suggested that intranasally administered TMZ16e-loaded NPs could effectively lengthen survival time, showing high anti-glioma activity. According to western blot analysis, reversing TMZ resistance was linked to the effective downregulation of the MGMT protein. The TMZ16e-loaded NPs were found to improve brain targeting efficiency and anti-glioma activity, as well as reverse TMZ resistance. Therefore, the TMZ16e-loaded NPs represent a promising approach for the treatment of drug-resistant glioma and provide an experimental basis for the clinical treatment of glioma.

## Data Availability

The original contributions presented in the study are included in the article/supplementary material, further inquiries can be directed to the corresponding author.
